# The Impact of Comorbidities on the Outcomes of Egyptian COVID-19 Patients: A Follow-Up Study

**DOI:** 10.1155/2021/6662476

**Published:** 2021-06-17

**Authors:** Reda M. Albadawy, Bismeen A. Jadoon, Mysara M. Mogahed, Mohamed E. Ibrahim, Tarek S. Essawy, Ahmed M. A. Amin, Marwa S. Abd-Elraouf, Mona A. Elawady

**Affiliations:** ^1^Department of Hepatology Gastroenterology and Infectious Diseases, Benha University, Faculty of Medicine, Banha, Egypt; ^2^WHO Consultant, Research Coordinator at ERC-RCOG, Giza, Egypt; ^3^Internal Medicine Department, Benha University, Faculty of Medicine, Banha, Egypt; ^4^Chest Department, Benha University, Faculty of Medicine, Banha, Egypt; ^5^Public Health Department, Faculty of Medicine, Benha University, Banha, Egypt

## Abstract

**Objectives:**

This study evaluated the clinical manifestation of COVID-19 and adverse outcomes in patients with comorbidities (outcome: death).

**Methods:**

A comparative follow-up investigation involving 148 confirmed cases of COVID-19 was performed for a month (between April and May 2020) at Qaha Hospital to describe the clinical characteristics and outcomes resulting from comorbidities. Participants were divided into two clusters based on the presence of comorbidities. Group I comprised cases with comorbidities, and Group II included subjects without comorbidity. Survival distributions were outlined for the group with comorbidities after the follow-up period.

**Results:**

Fever (74.3%), headache (78.4%), cough (78.4%), sore throat (78.4%), fatigue (78.4%), and shortness of breath (86.5%) were the most prevalent symptoms observed in COVID-19 patients with comorbidities. Such patients also suffered from acute respiratory distress syndrome (37.8%) and pneumonia three times more than patients without comorbidities. The survival distributions were statistically significant (chi-square = 26.06, *p* ≤ 0.001).

**Conclusion:**

Multiple comorbidities in COVID-19 patients are linked to severe clinical symptoms, disease complications, and critical disease progression. The presence of one or more comorbidities worsened the survival rate of patients.

## 1. Introduction

The Coronaviridae family has become the foremost microorganism triggering increasing disease outbreaks. It is a vast family of single-stranded RNA viruses (+ssRNA) that can be isolated from totally different animal species. These viruses can cross interspecies barriers and cause ill health in humans ranging from common cold to more severe respiratory diseases [1].

An ongoing public health emergency of international concern occurred due to an epidemic of COVID-19, which began in China in December 2019. Patients confirmed to have contracted this illness suffer outcomes ranging from mild to severe health issues to death. The incubation period of the disease is around 2–14 days after exposure. One or more subsequent symptoms could include (but not be restricted to) fever, cough, diarrhea, or labored breathing [2]. In January 2020, SARS-CoV-2 was believed to cause an outbreak of severe pneumonia; however, pneumonia is now well known to be a complication caused by COVID-19 [3]. COVID-19 has advanced with terrible speed since its discovery. The World Health Organization (WHO) declared a pandemic on 11 March 2020 [4]. By 15 April, more than 1,900,000 cases and 123,000 deaths had been assumed worldwide [5].

The clinical spectrum of COVID-19 spans asymptomatic or mild symptomatic as well as clinical conditions characterized by respiratory failure. Artificial breathing aids and life support in intensive care units (ICU) are mandated for multiorgan and systemic manifestations such as sepsis, septic shock, and multiple organ dysfunction syndromes (MODS) [6].

The Chinese Center for Disease Control and Prevention (CCDC) has classified the clinical manifestations of the disease according to severity: delicate, severe, and critical. Delicate patients do not contract pneumonia. Severe patients display dyspnea, respiratory rate ≥30 min, blood gas saturation (SpO_2_) ≤ 93%, lung infiltrates >50% within 24–48 hours and/or <300, PaO_2_/FiO_2_ ratio, or P/F. This last measure denotes the ratio between the forces per unit area of the gas, PaO_2_, and therefore indicates the proportion of gas provided (fraction of impressed gas, FiO_2_). Critical patients suffer respiratory failure, septic shock, and/or MODS [7]. Acute respiratory distress syndrome (ARDS) implicates a significant new-onset respiratory failure or worsening of the related and already identified respiratory image. Distinct varieties of ARDS are distinguished based on degrees of hypoxia [8].

The number of patients currently diagnosed with COVID-19 has multiplied dramatically; however, the connection between comorbidity and patients with COVID-19 remains unclear. Previously conducted studies have demonstrated that comorbidities could lead to a poor prognosis. Identifying the major risk groups is crucial to decision-making concerning anti-COVID-19 therapy. To date, only a few systematic reviews have comprehensively explored whether the presence of common comorbidities increases risks for COVID-19 patients risk or better guides clinical practices. However, multiple studies and systematic reviews have reported the link between single risk factors and the severity of COVID-19 [9].

This study aimed to describe the clinical characteristics and outcomes observed in patients diagnosed with COVID-19 in addition to comorbidities.

## 2. Methods

The research question probed whether comorbidities influenced the clinical characteristics and outcomes of COVID-19 patients.

The null hypothesis for the study read that COVID-19 patients with one or more comorbidity display clinical characteristics and outcomes identical to COVID-19 patients without comorbidities.

The alternative hypothesis for the study was COVID-19 patients with one or more comorbidity display different characteristics and outcomes from COVID-19 patients without comorbidities.

### 2.1. Study Design

A comparative follow-up study of one month was initiated from the time of diagnosis and was conducted between April and May 2020. One month is the mean period of hospital stay estimated by a pilot study performed on 37 COVID-19 patients (20% of the calculated sample size). The results of the pilot trial were not included in the outcomes of the present study.

### 2.2. Sampling Technique

Random cluster sampling was deployed to select Qaha Central Hospital from all quarantine hospitals for COVID-19 patients in Egypt.

### 2.3. Sample Size Calculation

This study utilized results obtained from Open Epi, version 3, open-source calculator—SSCC, for the data extracted from another Egyptian study [10]. A minimal sample size of 180 was indicated.

The study population included 185 patients with COVID-19 who fulfill the specified inclusion criteria before adjusting for age and sex, after which the tally came to 148.

A trained team of physicians accomplished the data collection from April to May 2020 according to the hospital's standard protocols.

### 2.4. The Inclusion Criteria

Stipulated for the study population, specified cases of COVID-19 were confirmed according to the real-time reverse transcriptase polymerase chain reaction (RT-PCR) test taken through nasal swabs. The patients were required to display one or more symptoms including fever, headache, cough, sore throat, sputum production, fatigue, shortness of breath (SOB), nausea, vomiting, diarrhea, nasal congestion, conjunctival congestion, myalgia, and arthralgia.

The patients were classified into two groups for comparison. Group I included COVID-19 patients diagnosed with one or more comorbidity including hypertension (HTN), coronary heart disease (CHD), cerebrovascular disease, diabetes (DM), chronic obstructive airway disease (COPD), chronic liver disease (CLD), chronic kidney diseases (CKD), and malignancy. Group II encompassed confirmed patients of COVID-19 without comorbidities. Patients in each group were further categorized into three subgroups according to age: <18 y, 18 ≤ 60, and ≥60 y. The primary measured outcomes included death or survival. Descriptive analysis was performed for factors such as demographic characteristics (age and gender). Demographic and clinical predictors were examined. Unadjusted relationships between both groups were determined. The results were later adjusted for age and gender through propensity score matching using IBM SPSS version 26 software (SPSS Inc., Chicago, ILL Company). Categorical data were presented as numbers and percentages and were analyzed using chi-square and Fisher's exact test, while quantitative data were expressed as mean ± standard deviation and evaluated using ANOVA (*F*-test). Kaplan–Meier was employed for survival analysis. All tests were 2-sided, and a *p* value of less than 0.05 was considered statistically significant.

## 3. Results

### 3.1. Presence of Comorbidity

Table 1 shows the prevalence of symptoms and the rate of complications experienced by patients classified into the two groups. Fever (74.3%), headache, cough, sore throat, fatigue (equally presented as 78.4%), and SOB (86.5%) were observed in patients designated to Group I, who also demonstrated statistically significant higher incidences of complications compared to those nominated to Group II.

### 3.2. Outcome of Hospitalization

Table 2 presents the outcomes including mortality, persistent hospitalized status, and discharge resulting from the hospitalization of COVID-19 patients. The 30-day mortality rate was higher for patients aged ≥60 years (57.1%) than those aged 18–60 years of age. The mortality rate was significantly higher for COVID-19 patients with a history of comorbidities such as asthma, COPD, DM, HTN, CHD, CLD, and CKD (28.6%, 14.3%, 42.9%, 100%, 28.6%, 14.3%, and 14.3%, respectively). The same group of patients evinced significantly higher incidences of clinical symptoms such as fever, conjunctival congestion, nasal congestion, headache, cough, sore throat, fatigue, SOB, nausea, vomiting, myalgia, and cyanosis (100%, 42.9%, 57.1%, 85.7%, 100%, 100%, 100%, 100%, 28.6%, 14.3%, 57.1%, and 14.3%, respectively).

The incidences of pneumonia (42.9%) and ARDS (100%) were significantly higher in patients with mortality outcomes on the 30^th^ day of admission (*p* ≤ 0.001). The same group exhibited a much greater incidence of ground glass appearance on CT scans in comparison with patients who remained hospitalized or were discharged at a later date (100%) (*p* ≤ 0.001). The CT scan results of the latter group of patients revealed a positive ground glass appearance, multiple lobe involvement, and consolidations. The incidences of these CT scan observations among the three groups of patients (mortality, persistent hospitalized status, and discharge) were significant (*p* ≤ 0.001, <0.001, and 0.005, respectively) and were computed at 100%, 71.4%, and 57.1%, respectively.

Multilinear logistic regression was performed to detect the predictors of mortality in COVID-19. It disclosed that the presence of one or more comorbidity was the preeminent cause of mortality. In order of predominance, the presence of comorbidities resulted in death (B (95% CI): 36.27 (2.53–3.14)), cyanosis (23.18 (0.034–0.39)), and SOB (22.43 (9.55–13.64)) in patients designated to Group I (Table 3).

**Figure 1:**
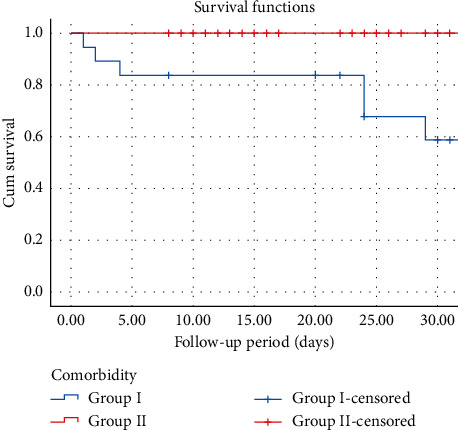
Survival curve of COVID-19 patients according to comorbidity presence.

Figure 1 illustrates the results of a log-rank test run to determine whether there were differences in the survival distribution of the two groups. The survival distributions were statistically significant (chi-square = 26.06, *p* ≤ 0.001).

## 4. Discussion

A vulnerable population, with chronic health conditions such as diabetes and cardiovascular or lung disease, is at higher risk of developing severe illness with COVID-19. This population is also faced with an increased risk of death in the event of illness [11]. Patients with comorbidities recorded significantly (*p*=0.001) higher percentages of complications, such as pneumonia and ARDS. This finding was congruent with the results reported by Bolin Wang et al., who revealed that patients with HTN (OR: 2.29, 95% CI: 1.69–3.10, *p* < 0.001), DM (OR: 2.47, 95% CI: 1.67–3.66, *p* < 0.001), or COPD (OR: 5.97, 95% CI: 2.49–14.29, *p* < 0.001) were at increased risk of infections from COVID-19 [9].

Another study also reported the relationship between CHD and patients with severe COVID-19 [12]. Furthermore, five studies comprising 313 severe group cases and 1167 nonsevere group cases evaluated the role of CLD in patients with COVID-19. The meta-analysis divulged that the history of CLD did not increase the risk of disease progression (OR: 0.67, 95% CI: 0.30–1.49, *p*=0.326) [13]. High blood pressure, dyslipidemia, diabetes, and obesity were marked as the main risk factors for poor prognoses because COVID-19 can increase blood glucose levels [14]. Some evidence indicates the arrhythmogenic potential of the drugs used in COVID-19 and moots the possibility that SARS-CoV-2 infection and other coronaviruses could cause arrhythmias [15]. The impact of underlying asthma, other allergic diseases, and T2 inflammation is suspected vis-à-vis susceptibility to COVID-19 and adverse disease outcomes. The better these interactions are understood, the more can the most vulnerable people be protected, including patients in high-risk groups [16].

Li et al. found that the levels of CRP, serum ferritin, and ESR (inflammation indicators) increased in COVID-19 patients and were associated with the greater severity of the disease. The levels of these indicators were higher in the patients with CVD than in those without CVD. This result indicates that COVID-19 exerted an additional impact on cases with a history of CVD [17].

Table 1 presents that the prevalence of symptoms in the current study is similar to the findings of Lechien et al. who reported the most common symptoms as headache (70.3%), nasal obstruction (67.8%), cough (63.2%), asthenia (63.3%), myalgia (62.5%), rhinorrhea (60.1%), and sore throat (52.9%). Fever was reported by 45.4% of the patients [18]. Another study testified that 82.4% of the patients suffered from cough, 64.8% from fever, and 38.5% from fatigue [19]. Fever was associated both with the development of ARDS and with better outcomes [20].

Dysregulated inflammation, compromised immune systems, and levels of ACE2 receptors in kidneys may explain disease severity and mortality-related outcomes in COVID-19 patients with a history of CKD. The mortality rate was found to be 53.33% and 17.65%, respectively, in COVID-19 patients with CKD and liver diseases [21].

This study reported a 30-day mortality rate of 17.8% due to COVID-19. This incidence is congruent with the assertion of a mortality rate of 13%, made by another study [22]. Richardson et al. reported age to be a risk factor for COVID-19. Age was also a factor that could help detect the outcomes of contracting COVID-19. Patients with DM or HTN who died and patients who had to be given intensive care ventilation or ICU care also belonged to older age groups [23].

### 4.1. Limitations of the Study

The current study admits to two limitations. First, only patients with relatively severe pneumonia due to COVID-19 were hospitalized during this period because of limited resources. Second, this study was conducted at a single hospital. The difference in population characteristics at various locations may produce different results.

## 5. Conclusion

In summary, the presence of multiple comorbidities in patients diagnosed with COVID-19 indicated significant symptoms and clinical manifestations and predicted a severe disease progression. The existence of more than two comorbidities worsened the survival rate of patients. A thorough assessment of comorbidities at the time of hospital admission may help establish the risk stratification of patients with COVID-19.

It is therefore suggested that a COVID-19 patient with one or more comorbidity may be saved if proper medical service is available. Prevention should be attempted by all possible means including vaccination and should be prioritized for high-risk populations and people with risk factors, especially chronic diseases. The management of a chronic ailment requires dietary modification, regular exercise, and adequate adherence to medications. The realities of isolation, social distancing, and lockdowns of cities pose many complex challenges for such individuals, significantly influencing their health and lifestyles.

## Figures and Tables

**Table 1:** Distribution of the studied groups according to comorbidities.

	Presence of comorbidity				*P* value
	Group I (with comorbidity) (74)		Group II (without comorbidity) (74)		
	*N*	(%)	*N*	(%)	
Symptoms					

History of fever	55	74.3	21	28.4	<0.001^*∗∗*^

Conjunctival congestion	20	27.0	2	2.7	<0.001^*∗∗*^

Nasal congestion	24	32.4	16	21.6	0.14

Headache	58	78.4	31	41.9	<0.001^*∗∗*^

Cough	58	78.4	46	62.2	0.031^*∗*^

Sore throat	58	78.4	43	58.1	0.008^*∗∗*^

Sputum production	4	5.4	1	1.4	0.37

Fatigue	58	78.4	30	40.5	<0.001^*∗∗*^

Shortness of breath	64	86.5	11	14.9	<0.001^*∗∗*^

Nausea	22	29.7	4	5.4	<0.001^*∗∗*^

Vomiting	12	16.2	2	2.7	0.005^*∗∗*^

Diarrhea	4	5.4	1	1.4	0.37

Myalgia	28	37.8	4	5.4	<0.001^*∗∗*^

Cyanosis	4	5.4	0	0.0	0.12

Complications					

Pneumonia	20	27.0	5	6.8	0.001^*∗∗*^

ARDS	28	37.8	0	0.0	<0.001^*∗∗*^

Outcome of hospitalization					
Died	28	37.8	0	0.0	<0.001^*∗∗*^
Survived	46	62.2	74	100	

Before any adjustment	*N* = (80)		*N* = (105)		
Gender					
Male	46	57.5	48	45.7	0.03^*∗*^
Female	34	42.5	67	54.3	

Age					
<18 y	0	0.0	12	11.4	0.002
≥18 y	80	100	93	88.6	

Outcome of hospitalization					
Died	28	35.0	0	0.0	<0.001^*∗∗*^
Survived	52	65.0	105	100	

After adjustment with gender^	*N* = (76)		*N* = (81)		
Gender					
Male	44	57.9	48	59.3	0.86
Female	32	42.1	33	40.7	

Age					
<18 y	0	0.0	12	14.8	<0.001^*∗∗*^
≥18 y	76	100	69	85.2	

Outcome of hospitalization					
Died	28	36.8	0	0.0	<0.001^*∗∗*^
Survived	48	63.2	81	100	

After gender and age adjustment^	*N* = (74)		*N* = (74)		
Gender					
Male	42	56.8	43	58.1	0.87
Female	32	43.2	31	41.9	

Age					
<18 y	0	0.0	3	4.1	0.08
≥18 y	74	100	71	95.9	

Outcome of hospitalization					
Died	28	37.8	0	0.0	<0.001^*∗∗*^
Survived	46	62.2	74	100	

^*∗*^Significant at *p* ≤ 0.05. ^*∗∗*^Significant at *p* < 0.01. ^Adjustment using propensity score matching. CT: computerized topography.

**Table 2:** Distribution of the studied groups according to outcome of hospitalization.

	Outcome of hospitalization (over 30 days)				*P* value
	Died (28)		Survived (120)		
	*N*	(%)	*N*	(%)	
Gender					
Male	16	57.1	69	57.5	0.97
Female	12	42.9	51	42.5	

Age					
<18 y	3	10.7	27	22.5	0.17
18 y	12	42.9	57	47.5	
≥60 y	13	46.4	36	30.0	

Symptoms					

History of fever	11	39.3	81	67.5	0.006^*∗∗*^

Conjunctival congestion	12	42.9	10	8.3	<0.001^*∗∗*^

Nasal congestion	16	57.1	24	20.0	<0.001^*∗∗*^

Headache	24	85.7	65	54.2	0.002^*∗∗*^

Cough	28	100	76	63.3	<0.001^*∗∗*^

Sore throat	28	100	73	60.8	<0.001^*∗∗*^

Sputum production	0	0.0	5	4.2	0.58

Fatigue	28	100	60	50.0	<0.001^*∗∗*^

Shortness of breath	28	100	47	39.2	<0.001^*∗∗*^

Nausea	8	28.6	18	15.0	0.089

Vomiting	4	14.3	10	8.3	0.30

Diarrhea	0	0.0	5	4.2	0.58

Myalgia	16	57.1	16	13.3	<0.001^*∗∗*^

Cyanosis	4	14.3	0	0.0	0.001^*∗∗*^

Complications					

Pneumonia	12	42.9	13	10.8	<0.001^*∗∗*^

ARDS	28	100	0	0.0	<0.001^*∗∗*^

^*∗*^Significant at *p* ≤ 0.05. ^*∗∗*^Significant at *p* < 0.01. CT: computerized topography.

**Table 3:** Multilinear logistic regression to detect predictors that cause death to COVID-19 patients.

	B	*P* value	Lower limit of 95% CI	Upper limit of 95% CI
Comorbidity presence (Group II is reference)	36.27	<0.001^*∗∗*^	2.53	3.14
History of fever	−1.4	0.19	−3.5	114.2
Headache	−3.37	0.08	0.0	96.45
Cough	−3.10	0.082	−11.43	2.15
SOB	22.43	0.004^*∗∗*^	9.55	13.64
Nasal congestion	4.06	0.074	0.0	121.87
Fatigue	4.11	0.07	−3.6	1.22
Cyanosis	23.18	0.001^*∗∗*^	0.034	0.39

^*∗∗*^Significant at *p* < 0.01. CI: confidence interval.

## Data Availability

The data used to support the findings of this study are available from the corresponding author upon request.
